# 24-h sheltering behaviour of individually kept horses during Swedish summer weather

**DOI:** 10.1186/s13028-015-0135-x

**Published:** 2015-08-20

**Authors:** Elke Hartmann, Richard J Hopkins, Claudia von Brömssen, Kristina Dahlborn

**Affiliations:** Department of Anatomy, Physiology and Biochemistry, Swedish University of Agricultural Sciences, Box 7011, 750 07 Uppsala, Sweden; Natural Resources Institute, University of Greenwich, Central Avenue, Chatham Maritime, Kent, ME4 4TB UK; Department of Ecology, Swedish University of Agricultural Sciences, Box 7044, 75007 Uppsala, Sweden; Unit of Applied Statistics and Mathematics, Department of Economics, Swedish University of Agricultural Sciences, Box 7013, 75007 Uppsala, Sweden

**Keywords:** Equine, Shelter, Behaviour, Weather, Insects

## Abstract

**Background:**

Provision of shelter for horses kept on summer pasture is rarely considered in welfare guidelines, perhaps because the benefits of shelter in warm conditions are poorly documented scientifically. For cattle, shade is a valued resource during summer and can mitigate the adverse effects of warm weather on well-being and performance. We found in a previous study that horses utilized shelters frequently in summer. A shelter with a roof and closed on three sides (shelter A) was preferred and can reduce insect pressure whereas a shelter with roof and open on three sides was not utilized. However, shelter A restricts the all-round view of a horse, which may be important for horses as flight animals. Therefore, we studied whether a shelter with roof, where only the upper half of the rear wall was closed (shelter B), would be utilized while maintaining insect protection properties and satisfying the horses’ sense for security. A third shelter was offered with walls but no roof (shelter C) to evaluate whether the roof itself is an important feature from the horse’s perspective. Eight Warmblood horses were tested each for 2 days, kept individually for 24 h in two paddocks with access to shelters A and B, or shelters A and C, respectively. Shelter use was recorded continuously during the night (1800–2400 h, 0200–0600 h) and the following day (0900–1600 h), and insect defensive behaviour (e.g., tail swish) in instantaneous scan samples at 5-min intervals during daytime.

**Results:**

Seven horses used both shelters A and B, but when given the choice between shelters A and C, shelter C was scarcely visited. There was no difference in duration of shelter use between night (105.8 ± 53.6 min) and day (100.8 ± 53.8, *P* = 0.829). Daytime shelter use had a significant effect on insect defensive behaviours (*P* = 0.027). The probability of performing these behaviours was lowest when horses used shelter A compared to being outside (*P* = 0.038).

**Conclusions:**

Horses only utilized shelters with a roof whilst a shelter with roof and closed on three sides had the best potential to lower insect disturbance during daytime in summer.

## Background

Studying the benefits of providing man-made shelters for horses during the summer months has until recently received little scientific attention. This topic has been thoroughly addressed in other livestock, such as dairy cattle, presumably due to the direct positive effects the provision of shade has on productivity [1–3].

Individually housed horses studied by Holcomb et al. [4] used shade when given the choice during hot, sunny weather. Being in shade under a shelter structure increased feeding behaviour and locomotion and did not alleviate physiological changes that may have otherwise occurred in response to lack of shade [5]. Furthermore, the provision of a man-made shelter may benefit horses because it can lower insect harassment [6, 7]. Diminishing insect pressure by seeking refuges with low insect activity (e.g., open spots with sparse vegetation and higher wind velocities) can take precedence over seeking shade for horses [8–10]. Thus, natural shelter, such as forest, may not provide sufficient protection from severe insect attacks. Polish Konik horses, for example, performed more frequent insect defensive behaviours when kept in a forest area compared to being kept on open pastures during summer [11]. Vegetation may provide good microhabitats for insects to rest, leading to a high insect density in areas with high densities of trees and bushes [12]. Blood-sucking insects are likely to present a cost to the animal as they cause animals to change habitat and behaviour to minimise irritation and there is the possibility of transmitting infectious diseases via bites, or inducing allergies [13].

Although shade, provided by a shelter with a roof and open on all four sides benefited horses in physiological terms, this shelter layout seemed insufficient to lower insect avoidance behaviour [4, 5]. A shelter with closed sides may give better protection because it becomes more difficult for insects to visually locate the horse.

The purpose of the current study was to evaluate whether individually kept horses exhibit preferences for a specific shelter structure and to determine which of the provided shelters has the potential to lower insect harassment during daytime; a shelter with roof and closed on three sides, a shelter with roof and partially closed on the rear wall or a shelter without roof and three closed walls. Furthermore, it was aimed to determine whether shelter use was related to weather conditions and to what extend horses would make use of shelters during the night.

## Methods

### Horses and management

The current study was conducted from the end of June until mid-July in 2013 and was a follow-up to a study conducted in July 2012 at Jälla Agricultural High School in Uppsala, Sweden. Thus, the study site and experimental methods for the current study were the same as described previously by Hartmann et al. [6]. Six of the eight horses (5 mares, 1 gelding) were also used in the previous study whilst two geldings were naïve to the current study design. All horses had a dark coat colour (chestnut, bay). They were accustomed to both frequent handling and to individual turnout in paddocks and to being stabled individually in boxes during the night.

When no testing was taking place, horses were kept for 24 h in groups on pasture with access to natural shelter (trees, bushes) and grassland. Water was available ad libitum and no supplementary feed was provided. During testing, haylage was offered at 0800, 1200 and 2000 h (3–4 kg per feeding) outside the shelters next to the paddock entrance because grass was sparse. Water was available ad libitum from pressure valve bowls.

### Study design and data collection

All eight horses were habituated to the test paddocks and shelters prior to the start of this study. They were each led by one person in and out of the shelters several times during four 15-min sessions per horse spread over 2 days. The habituation criterion was met when shying and attempts to leave the shelter diminished and horses could remain calmly inside the shelter together with the person for 5 min.

**Fig. 1:**
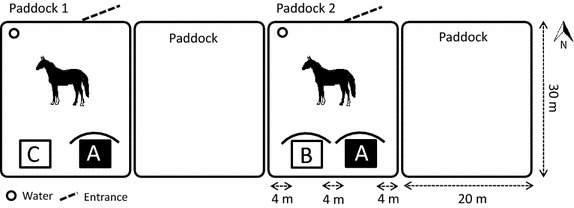
Horses were kept individually in paddocks 1 and 2 during two test days with access to shelters A and B, and shelters A and C, respectively. The rear sides of the shelters were placed next to the fence so that horses could not pass behind. No shade other than from shelters was available throughout daytime. No horses were kept in the adjacent paddocks.

**Fig. 2:**
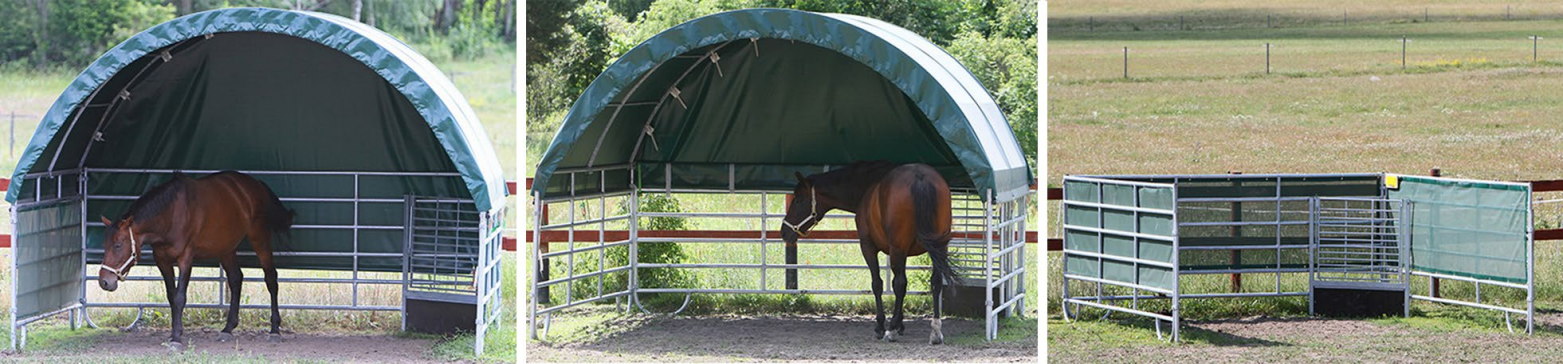
Closed shelter A (*left* with opaque plastic roof, opaque plastic on the rear wall opposite the entrance and transparent wind nets on two sides, open shelter B (*middle* with opaque plastic roof and opaque plastic covering the upper half of the rear wall), and shelter C without roof (*right* wind nets on three sides). Shelters were purchased from Mobile Covers (Cover all Europe GmbH, Groß Lüdershagen, Germany) and measured 4 × 4 m (height 3.15 m). The fence elements consisted of 2 mm thick round steel (45 mm in diameter) and the distance between fence elements was 21.5 cm (*first bar* at 23 cm off the ground). The height of the walls measured 130.5 cm. The roof was a polyvinyl chloride fabric (670 g/m^2^). Sticky paper traps were placed in the right corner in each shelter behind a metal gate.

The horses were tested in pairs, and these pair constellations were kept the same throughout the study. During testing, each horse of the pair was kept individually in a paddock (Fig. 1) during 2 days with access to shelters of three different layouts: (a) closed shelter with an opaque plastic roof, opaque plastic on the rear wall opposite the entrance and transparent wind nets on two sides, (b) open shelter with opaque plastic roof and opaque plastic covering the upper half of the rear wall, and (c) open shelter without roof and wind nets on three sides (Fig. 2). The horses were kept in the paddocks from 1600 to 1600 h the following day.

Shelter use was defined as a horse standing with at least two hooves inside the shelter. It was recorded continuously (in min) during the evening, night and early morning from video recordings between 1800 to 2400 h and 0200 to 0600 h (surveillance camera, Qihan Technology Co., Ltd., China) and during daytime from 0900 to 1200 h and 1300 to 1600 h by two observers sitting outside the paddocks at a distance of 30 m. Other behaviour, including shelter use was recorded via direct observations at 5 min intervals according to the ethogram in Table 1.

**Table 1:** Ethogram of behaviours

Behaviour	Description
Stand	Standing inactive with head lowered or elevated, can include one hind leg flexed
Feed	Ingest grassy vegetation or haylage
Insect defensive behaviour (comfort behaviour)	
Groom	Nibbling, biting, licking or rubbing a part of the body
Shake	Rapid rotation of the head, neck and upper body while standing
Swat	Swing of head against the shoulder or abdomen, flex the chin to the chest
Stomp	Sharply strike the ground by rapidly flexing a fore or hind leg
Skin shiver	Rapid twitching of the skin at the withers
Ear flick	Rapid rotation of one or both ears without moving the head
Tail swish	Swishing of the tail from its resting position to one side or up and down

Rectal temperature (RT; digital thermometer, Flex Temp Smart, Omron healthcare Co. Ltd., Kyoto, Japan) and skin temperature (ST; thermistor probe, Ellab, Hillerød, Denmark) were measured at 0800, 1200 and 1600 h. ST was measured on two shaved spots (5 × 5 cm) on the horse’s left neck (halfway from head to withers) and left hindquarter (halfway between hip and buttock). Respiration rate (RR) was recorded before measurement of RT and ST by counting flank movements during 15 s and the average was calculated for 1 min.

Insect activity was monitored by catching winged insects (mostly true flies Diptera) on yellow sticky paper traps (10 × 25 cm, Catch-it™, Silvandersson Sweden AB, Knäred, Sweden) placed in the right, rear corner of each shelter opposite the entrance. As a control, one paper trap was placed 100 m away from horses on an open spot on grassland. Sticky papers were replaced twice daily, in the morning (night catch) and afternoon (day catch).

Weather conditions were registered at 10 min intervals with a weather station (Vantage Pro2, Davis Instruments, Hayward CA, USA) including ambient temperature (Ta), relative humidity (RH), and wind speed (WS). Due to technical problems, solar radiation was not recorded at the study site but it was documented at every sampling point whether the sun was visible or was covered with clouds (cloudiness). To measure microclimate in the shelters, two temperature sensors were placed in the shelters A and B, 30 cm below the roof (Hobo Data Loggers, Onset Computer Corporation, Bourne, MA, USA). The temperature humidity index (THI) was calculated based on the formula by Thom [14]: THI = (0.8 × ambient temperature) + {[(relative humidity/100) × (ambient temperature − 14.4)] + 46.4}.

### Data analyses

The Generalized Linear Mixed Model (GLMM with Proc Glimmix, binary distribution) was used for modelling the probability of horses being observed outside the shelters in relation to Ta and WS recorded outside shelters. The model was run with each weather variable as a separate covariate. The same approach was taken for modelling the effect of the THI index and the number of flies caught in the control trap. The individual effect of RH was not tested as the possible combined effect of RH with Ta was considered in the THI index. It was avoided to test interaction parameters between weather variables (e.g., between Ta and RH) because of the expected correlations between them, making the interaction estimation unstable. The GLMM was also used for comparing the insect defensive behaviour “tail swish” and “standing” between horses that were observed either in shelter A, B or outside. Only data from horses kept in paddock 2 were used for this analysis because shelter B was not available in paddock 1. Data followed a binomial distribution which was accounted for in the model. Since the number for the remaining insect defensive behaviours was too low for analysis, a new variable called “defense” was created, consisting of the sum of the behaviours groom, shake, swat, stomp, skin shiver, and ear flick. The same model was used but assuming a Poisson distribution.

The Generalized Linear Model (GLM with Proc Mixed) was applied for testing whether the duration (in min) horses used shelters A and B differed between night and day. The duration of shelter use was transformed into proportions because of the different length of night (total 10 h) and day (total 6 h). This model was also taken to test the effect of shelter use on the physiological parameters RT, ST and RR. For that purpose, a new variable was created and assigned to a horse when it was observed for at least 30 min continuously inside the shelters with roof immediately before measurement at 1200 and 1600 h.

Differences in Ta, RH and THI between the two shelters with roof and outside were assessed with the parametric Two-sample *t* test. The Chi square goodness-of-fit test was used to test whether the number of flies caught in the three shelters and the control trap differed. The relationships between numbers of flies caught in the control trap during daytime and weather variables, including THI index for outside shelters were tested with Spearman’s rank correlation.

Results from the models are presented as least square means with standard error. Other results are reported as mean with standard deviation. The significance level was set at *P* < 0.05. Data were analysed in the statistical software SAS (Version 9.3, SAS Institute Inc., Cary, NC, USA).

## Results

### Weather conditions and insect activity

The average Ta during the study period was 19.2 ± 2.1 °C during daytime and 14.5 ± 2.5 °C during the night (Table 2). During daytime, it was significantly warmer in shelter A and shelter B compared to outside, mirrored in a 5.8 °C (25.0 ± 3.9 °C, Two-sample t-test: t = −3.75, *P* = 0.004) and 3.9 °C (23.1 ± 3.0 °C, t = −3.03, *P* = 0.010) temperature difference between outside and Shelters A and B, respectively. The same pattern was observed for RH (shelter A: 39.8 ± 10.1 %, t = −3.55, *P* = 0.004; shelter B: 42.8 ± 9.5 %, t = −3.02, *P* = 0.010; outside: 56.7 ± 9.0 %) and THI (shelter A: 70.4 ± 3.8, t = 3.76, *P* = 0.003; shelter B: 68.4 ± 3.1, t = 2.88, *P* = 0.013; outside: 64.3 ± 2.5) during daytime. No differences in Ta, RH and THI between locations were detected during the night (*P* > 0.05). WS did not exceed 4.0 m/s during the day and was at a maximum 2.5 m/s during the night. Cloudiness was scored during 34.2 % of daytime observations (394 out of 1,152 observations).

**Table 2:** Mean ± SD of ambient temperature (Ta), relative humidity (RH), and wind speed (WS) recorded during the night (1800–0600 h) and the following day (0900–1600 h)

Test day	Ta (°C)		RH (%)		WS (m/s)	
	Night	Day	Night	Day	Night	Day
1	13.7 ± 3.6	19.7 ± 1.2	86.1 ± 12.1	58.5 ± 8.8	0.2 ± 0.3	1.6 ± 3.2
2	15.6 ± 3.1	17.3 ± 0.6	72.3 ± 11.2	68.4 ± 7.3	1.0 ± 0.5	2.9 ± 0.4
3	13.9 ± 2.5	18.5 ± 1.2	70.3 ± 12.4	50.5 ± 5.1	0.7 ± 1.0	2.1 ± 0.5
4	15.2 ± 3.0	19.2 ± 1.3	68.8 ± 14.6	67.7 ± 7.7	0.7 ± 0.5	1.0 ± 0.4
5	19.8 ± 3.8	17.8 ± 1.4	61.2 ± 9.4	49.2 ± 3.8	1.7 ± 0.8	1.8 ± 0.3
6	12.6 ± 4.1	23.0 ± 1.7	73.5 ± 15.8	44.3 ± 6.8	0.4 ± 0.7	3.2 ± 0.5
7	13.6 ± 2.3	16.8 ± 0.8	74.4 ± 9.1	62.7 ± 5.0	0.9 ± 0.5	1.7 ± 0.3
8	11.5 ± 4.4	21.0 ± 1.8	78.6 ± 16.4	52.6 ± 6.6	0.3 ± 0.4	1.1 ± 0.4
Mean	14.5 ± 2.5	19.2 ± 2.1	73.2 ± 7.3	56.7 ± 9.0	0.7 ± 0.5	1.9 ± 0.8

The number of flies caught on the sticky paper traps during daytime differed significantly between trap locations (Chi squared: χ^2^ = 10.8, df = 3, *P* = 0.013). Most flies were caught during the day in the control trap (total 13 during study period), and in shelter A and C (total 7 and 6, respectively), and least flies in shelter B (total 1). During the night, fly activity was low outside the shelters (total 4), in shelter A (total 1) and shelter C (total 6), and no flies were caught in shelter B.

There was a significant negative correlation between the variable cloudiness and number of flies caught in the control trap during daytime (r = −0.165, *P* = 0.001). The correlations between the remaining weather variables and the number of flies were not significant (Ta: r = 0.345, *P* = 0.403; RH: r = −0.115, *P* = 0.786; WS: r = −0.077, *P* = 0.857; THI: r = 0.345, *P* = 0.403).

### Shelter use and effect of weather and insects

**Fig. 3:**
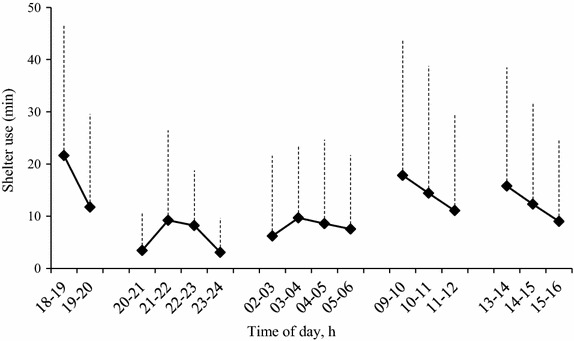
Hourly mean duration (SD) seven horses were observed inside shelter A and shelter B. No recordings were made between 2400 and 0200 h because of darkness. Haylage was provided at 2000, 0800, and 1200 h.

Seven of the eight horses used the shelters (Table 3). They were observed inside the shelters during 35.4 % of daytime (0900–1200 h and 1300–1600 h) observations during the study period (408 out of 1,152 observations), and on average during 34.5 % of daytime observations per test day (51 out of 148 observations). The time (in min) spent inside the shelters A and B with roof did not differ significantly between night (105.8 ± 53.6 min) and day (100.8 ± 53.8 min, *P* = 0.829). Shelter A was visited less during nights (53.9 ± 53.8) compared to shelter B (157.8 ± 57.3, *P* = 0.006) but there was no difference in duration during daytime (P = 0.341). Horses were observed inside the shelters on average longest between 1800 and 1900 h and between 0900 and 1000 h the following day (Fig. 3).

**Table 3:** Total duration (in min) eight horses were observed during night (1800–2400 h, 0200–0600 h) and day (0900–1200, 1300–1600 h) in the two paddocks (P1, P2) inside the shelters A, B, and C

Horse	Shelter A				Shelter B		Shelter C	
	P1		P2		P2		P1	
	Night	Day	Night	Day	Night	Day	Night	Day
Adina	26	92	93	10	284	72	2	0
Armangac	2	10	1	0	0	4	1	1
Bengan	177	254	22	0	112	290	0	2
Calypso	273	213	36	7	262	145	9	0
Cortina	88	37	5	2	66	9	1	8
Colette	0	0	0	0	0	0	0	0
Rizzo	59	90	4	58	20	66	12	1
Tanja	176	357	22	1	144	0	25	0
Mean	100.1	131.6	22.9	9.8	111.0	73.3	6.3	1.5

The probability of observing horses outside shelters during daytime was not affected by Ta recorded outside shelters, WS, THI index or the number of flies in the control trap (*P* > 0.05). A cloudy sky (cloudiness) tended to increase the probability of observing horses outside shelters, although not statistically significant (*P* = 0.058).

Tail swishing was the most frequently performed insect defensive behaviour in horses while kept in paddock 2 but was not related to shelter use (*P* = 0.072, Table 4). Yet, the probability of tail swishing was smallest when horses were observed inside shelter A compared to outside (*P* = 0.029, Table 4). The probability of horses performing other insect defensive behaviours summarized under the variable “defense” were lowest when horses were using shelter A (*P* = 0.038) and tended to be lower for shelter B than when horses were observed outside shelters (*P* = 0.060, Table 4).

**Table 4:** Modelled insect defense (mean number of activity per 5 min, ±SEM), tail swishing, and standing (probability of activity in %, ±SEM) when horses were observed inside shelter A, B, and outside

Behaviour	Shelter A	Shelter B	Outside	Den DF	F-value	P-value
Defense^A^	0.04 ± 0.03^a^	0.11 ± 0.04^ab^	0.20 ± 0.06^b^	573	3.62	0.027
Tail swish^B^	6.6 ± 5.4^a^	16.6 ± 9.2^ab^	23.0 ± 10.8^b^	558	2.65	0.072
Stand^B^	80.0 ± 12.2^a^	96.9 ± 1.7^b^	50.8 ± 8.0^a^	558	22.1	<0.001

^a, b^Between columns, numbers without a common superscript differ significantly (*P* < 0.05).

^A, B^Results were obtained from two separate models.

### Shelter use in relation to physiological parameters

Five of the eight horses fulfilled the criterion of standing inside the shelters for at least 30 min before measurement whereby shelter A was used exclusively. Neither RT, ST nor RR differed by time of the day between horses using the shelter and those standing outside (*P* > 0.05).

RT increased during the day from 37.4 °C (SD = 0.3) measured at 0800 h to 37.5 °C (SD = 0.2) at 1600 h. ST of the neck was 34.0 °C (SD = 0.7) in the morning and 35.6 °C (SD = 1.1) in the afternoon, and ST of the rump 33.1 °C (SD = 1.3) and 36.0 °C (SD = 1.9), respectively. Average RR was 16 breath per minute (SD = 3.1) at 0800 h and 17.7 (SD = 6.0) at 1600 h.

## Discussion

Individually kept horses used shelters with a roof during both the night and the day. The horses preferred shelters with a roof and partially closed on at least one side (shelter B) or three sides (shelter A) when these shelters were tested in combination. When given the choice between shelter A and a shelter closed on three sides but without a roof (shelter C), the shelter with a roof (A) was favoured. Noticeably, shelter use reflected individual preferences as some of the horses hardly used any of the provided shelters which were also the same individuals studied in the previous experiment [6].

Shelter use during daytime comprised 34.5 % of observations in the current study which was similar to results obtained from the previous shelter study conducted during 2 weeks in summer of 2012 (shelter use 29.1 %) [6]. This was a lower percentage of shelter use compared to that reported by Holcomb et al. [4], possibly related to the differences in paddock size and the corresponding area that was occupied by shelters. Their horses were studied under arid and hot conditions in California where horses were standing beneath a shelter structure covering half of the pen (6.1 × 6.1 m) greater than chance in 10.3 % of observations during daytime (preference for structure use was calculated as the difference between total use and chance, the latter corresponding to 50 %) [4]. Yet, even warmer weather (mean daytime ambient temperature 29 ± 5 °C) did not seem to pose a thermal challenge to these horses as they were able to maintain RT, SK and RR throughout the day by having the option to seek shade [4]. The lack of differences in these physiological measures in the present and also in the previous study [6] is highly likely related to the moderate weather conditions (mean daytime ambient temperature 19.2 ± 2.1 °C and 19.7 ± 1.0 °C, respectively), not posing a thermal challenge to mature, healthy horses.

There is a substantial body of evidence showing that cows use shade significantly more often when weather becomes warmer [15, 16], and that they prefer shade that provides higher levels of protection from solar radiation (shelter cloth blocking 50 and 99 % of solar radiation versus 25 %) [16, 17]. Results from a recent study by Holcomb et al. [4] showed that horses were located in the shade especially before and during peak solar radiation. Under the prevailing weather conditions, Ta did not affect shelter use but results revealed that horses tended to use the shelters less the cloudier it was. The amount of solar radiation absorbed by the coat and its reflective properties partly determine the heat load experienced by an animal [18]. Coat colour is one characteristic that determines the impact of solar radiation. Dark coloured coats usually absorb more solar radiation than light coloured coats and would thus increase heat load of dark coloured animals [15]. All the horses in our study had dark coat colours (chestnut, bay) but whether the pattern of shelter seeking behaviour would differ significantly between individuals of different coat colour can only be speculated. What is evident is that blood-sucking flies are more attracted to dark coloured coats because of the polarizing characteristics of the body surface [19]. Reducing annoyance from insects may thus be more important than seeking shade given the moderate summer weather during the study period and the finding that insect defense was lower in horses using particularly the closed shelter A.

How weather is experienced certainly depends upon a combination of different weather variables [20, 21]. Yet, testing combinations of weather variables on shelter use has been avoided. This was decided because of the expected correlations between weather variables which would have made the interaction estimation in the model unstable. The analysis of the THI index has been used for decades to assess the effect of weather on livestock [3] although it has limitations because other factors such as wind speed, which may have a cooling effect, are not taken into account [21]. Furthermore, heat stress classes may not be directly applicable to horses in comparison to high producing cattle for which the index was originally established, given differences in behaviour, physiology and body type. The THI index calculated for conditions outside shelters during daytime was on average 64.3. This was within a normal range (<74 THI) of the defined heat stress classes and the critical values reported to have negative effects on the physiology and production of dairy cattle were never reached in the current study [3]. Thus, the ambient conditions during the experimental period were certainly within the thermoneutral zone of horses which can range from −7 to 30 °C, depending on season, region, breed and/or age [20].

The microclimate in the shelters A and B with a roof was generally warmer than outside which was also the personal experience of the authors. It may be possible that horses experienced the outside ambient temperature as relatively cool and therefore sought thermal comfort in the warmer shelters. Thus, shelters have perhaps not served for cooling even though wind nets on two sides and a partially open rear wall allowed some airflow. van Laer et al. [3] and Blackshaw and Blackshaw [15] have summarized that the effectivity of shelters in terms of reducing heat load largely depends upon the material used and the structure of the shelter. Tucker et al. [17], for example, used open sided shelters under which Ta was below outside conditions. Yet, the authors pointed out that it is difficult to interpret whether two degree temperature difference would be experienced as cool from the animal’s point of view. Furthermore, the addition of one wall can affect the radiant heat load, whereby a three-sided shelter has been shown to reduce most radiant heat [22]. Contrary to this, the dark green colour and the material of the roof (polyvinyl chloride fabric) of the shelters in our study may have accumulated more heat than a light colour or other material would have done [15].

Shelters were frequently used during nights and it seems that some feature of the shelter structure was appealing beside the possibility that horses sought shelter to find shade. This may be due to an increased sense of security as was suggested by Holcomb et al. [4]. We propose that this may be relevant for singly kept horses that also have the experience of being stabled in boxes at night. During the cold season, horses seem to use shelters mostly during nights, and lying behaviour occurred almost exclusively inside the shelter [23, 24] which may support this security seeking hypothesis.

Another plausible explanation of shelter use may be that horses seek to avoid insects. Under free ranging conditions, horses often seek refuges at times of peak insect activity by moving to spots with maximum wind velocity, avoiding areas with dense vegetation [10]. Notably, many insects also rest in scrub and forest margins, making these areas less favourable for potential hosts [3]. Blood-sucking insects usually find their hosts initially via olfactory stimuli. When getting closer, visual contact is made whereby the host will be more easily detected the greater the contrast is with the background and the larger the animal [3]. Given these host searching strategies, it is possible that horses staying inside shelters, where at least one side is partially covered, become less apparent and harder to detect relative to the background. This may be supported by the finding that insect defensive behaviours were exhibited less in horses using shelters A and B. Tail swishing, in particular, tended to be lower in horses observed in the shelter closed on three sides (A) compared to the shelter with only one side partially closed (B). This confirms the findings from the previous shelter study [6], where the insect defensive behaviours skin shiver and ear flick were performed less frequently by horses standing beneath a three-sided shelter with a roof. In another study, no differences in insect defensive behaviour were found between horses completely shaded or unshaded [5]. This may have been due to the lack of walls, making it potentially easier for biting insects to find their hosts. Thus, the differences in insect defensive behaviours we found in the current study may be explained by the wall in the shelter structure blocking visual stimuli for searching flies. The differences in the number of flies caught on the sticky paper traps reflect this finding although the numbers were generally low compared to other studies [4, 5] which could be related to the method of catching [3, 25]. Insect defensive behaviours are effective physical attempts to reduce annoyance from insects landing and settling on the animal [13]. The degree of insect defensive behaviour is a reliable measure because it is directly related to the number of insects attacking the host [8, 26, 27].

Future research should establish whether horses kept in groups would seek shelter to a similar extent as when kept individually. If horses are kept in groups, it is likely that shelter use would be affected depending on social relationships between group members whereby horses higher in rank often have priority access [28]. Furthermore, studies have demonstrated that the number of fly attacks on an individual is reduced with increasing number of animals [27, 29–31]. This aggregation behaviour serves as a defence mechanism against temporary invasions of blood-sucking insects whereby animals in the periphery of the herd are usually at a disadvantage [3]. The positioning of shelters may also bias shelter seeking behaviour, for example, if placed in close proximity to water and feed troughs [3] or at locations causing visual obstruction from neighbouring conspecifics. Since horses are highly gregarious animals, realising group cohesion even when kept singly on separate paddocks may be important. The location of shelter A in paddock 2, for instance, may explain the difference in use compared to when the same shelter was available in paddock 1.

The current study measured the short term choices singly kept horses would make when kept on paddocks during summer. It needs to be pointed out that this may not reflect the motivational priorities an individual would establish in another context, as the choice may be affected by the length of exposure to the resource [32]. Therefore, future research on shelter use of horses kept 24 h on summer pasture would benefit from studies conducted over a longer period of time, covering a wider range of summer weather conditions. Focus may also be put on technical modifications of shelters such as using transparent curtains in the entrance area and using more reflective material to reduce insect harassment and heat load to a minimum.

## Conclusions

Access to shelter appears to be a valued resource for most horses when kept individually on paddocks during the summer. Shelter use may not be primarily related to weather conditions but is most likely dependent upon individual preferences. Providing shelter that is closed on three sides has the best potential to give some relief from flying insects and may therefore be taken into consideration in recommendations on the management of horses during summer.
